# Comparison of the combined use of CNV-seq and karyotyping or QF-PCR in prenatal diagnosis: a retrospective study

**DOI:** 10.1038/s41598-023-29053-6

**Published:** 2023-02-01

**Authors:** Hao Zhang, Zhihong Xu, Quan Chen, Huijuan Chen, Xiaoli Ding, Lin Liu, Yuanyuan Xiao

**Affiliations:** 1 Department of Reproductive and Genetic Diseases, Deyang People’s Hospital, Taishan North Road #173, Deyang, 618000 Sichuan Province China; 2 Deyang Key Laboratory of Birth Defects Prevention and Control, Deyang People’s Hospital, Taishan North Road #173, Deyang, 618000 Sichuan Province China

**Keywords:** Reproductive disorders, Disease prevention, Public health

## Abstract

To elevate the accuracy of diagnostic results, CNV-seq is usually performed simultaneously with karyotyping or QF-PCR. Although several studies have investigated the performance of the combined use of CNV-seq with karyotyping or QF-PCR, there have been no reports focusing on the comparison of these 2 diagnostic strategies. In our study, 2507 pregnant women were included to investigate these 2 strategies. The detection rates of foetal genetic abnormalities and turnaround time were compared between these 2 groups. Moreover, the detection rates of foetal genetic abnormalities in different indications were analyzed. Our results unveiled that the detection rates of numerical chromosomal abnormalities were nearly the same in these 2 groups. In addition to numerical chromosomal abnormalities, 39 balanced karyotypic changes and chromosome polymorphisms were detected via the combined use of karyotyping and CNV-seq. Further investigation revealed that the vast majority of these karyotypic changes were inherited from parents. Compared with the karyotyping group, the combination of QF-PCR and CNV-seq reduced the reporting time from 31.593 ± 4.944 days to 11.460 ± 4.894 days. Meanwhile, NIPT, maternal serum screening and ultrasound scan significantly improved the detection of foetal genetic abnormalities. In conclusion, our results revealed that parental karyotyping is a useful supplementary method for CNV-seq and systematic prenatal examinations improved the detection of foetal genetic defects.

## Introduction

According to the World Health Organization, birth defects affect 4–8% of births worldwide, and their incidence varies between different countries^1^. In China, the incidence of birth defects is approximately 5.6%^2^. Birth defects usually lead to foetal death, perinatal death, infant death or child disabilities. Chromosome aberrations, including aneuploid, triploid and deletion or duplication of large chromosome segments, are a major cause of birth defects^3,4^. Additionally, copy number variant syndromes (CNV syndromes), which are caused by pathogenic copy number variations (pCNVs), lead to intellectual disability, multiple congenital anomalies, autistic spectrum disorders and other diseases^5^. Due to a lack of cures for most birth defects caused by genetic abnormalities, termination of pregnancies with a prenatal diagnosis of foetuses with genetic aberrations is the primary response^6^.

Copy number variation sequencing (CNV-seq), based on high-throughput sequencing, was developed in 2009^7^. Through whole genome low-coverage sequencing, CNV-seq is able to detect genetic aberrations, including chromosome aneuploidies and CNVs larger than 100 kb^8^. Combined with the advantages of using small amounts of genomic DNA and detecting low-level mosaicism, CNV-seq has been used increasingly widely for prenatal diagnosis^9–12^. However, due to its low sequencing depth, CNV-seq cannot detect balanced karyotypic changes and polyploidies such as 69, XXX^8^. Additionally, maternal cell contamination affects the accuracy of CNV-seq and may lead to misinterpretation of testing results. Therefore, other detection methods need to be used in combination with CNV-seq for prenatal diagnosis.

Currently, CNV-seq is usually performed in combination with karyotyping or QF-PCR for prenatal diagnosis^9,11,13^. Karyotyping, the gold standard for detecting chromosome abnormalities, has been used for prenatal diagnosis since the 1960s^14^. With a resolution of approximately 5–10 Mb, karyotyping is able to detect karyotypic abnormalities, euploidies and abnormalities involving large chromosomal segments^15^. CNV-seq performed simultaneously with karyotyping is able to detect balanced karyotypic abnormalities, chromosomal polymorphisms and numerical chromosomal abnormalities^13^. However, the combined use of CNV-seq and karyotyping in prenatal diagnosis is labor-intensive and has a long turnaround time. Quantitative fluorescent polymerase chain reaction (QF-PCR) has been used as a rapid prenatal detection method for common aneuploidies and euploidies since 1999 by analyzing highly polymorphic short tandem repeats^16^. Moreover, maternal cell contamination, a potential hazard to the accuracy of CNV-seq, can also be identified by QF-PCR^17^. In 2019, experts recommended the combined use of CNV-seq and QF-PCR for prenatal diagnosis in China^18^. However, the combined use of CNV-seq and QF-PCR could not detect balanced karyotypic changes. Taken together, the combined use of CNV-seq and karyotyping and the combined use of CNV-seq and QF-PCR have their advantages and disadvantages in prenatal diagnosis. Until recently, no studies have focused on the comparison of these 2 diagnostic strategies. In our study, the detection rates of different abnormalities and turnaround time of pregnant women accepting prenatal diagnosis by CNV-seq and karyotyping or QF-PCR were analyzed. Moreover, we also analyzed the abnormalities detected in pregnant women with different indications. Our results revealed that parental karyotyping is a useful supplementary method of CNV-seq for the first time. Meanwhile, systematic prenatal examinations, including NIPT, maternal serum screening and ultrasound scans, helped to detect chromosome abnormalities and pCNVs.

## Results

### Clinical characteristics

A total of 2507 participants were included in our study. Of these, 2019 and 488 were included in the karyotyping group and QF-PCR group, respectively. Although the maternal age and gestational age of these 2 groups were not significantly different, the difference in indications between the karyotyping and QF-PCR groups was statistically significant (Table 1). Advanced maternal age and increased maternal serum screening risk were more common in the QF-PCR group, while ultrasound abnormalities and increased NIPT risk were more common in the karyotyping group.

**Table 1:** Clinical characteristics of pregnancies in the karyotyping and QF-PCR group.

	Karyotyping group	QF-PCR group	P value
Maternal age (years)	30.606 ± 5.537^a^	30.861 ± 5.435^a^	0.360
Gestational age (weeks)	20.820 ± 3.104^a^	20.699 ± 4.038^a^	0.535
Pregnancies	2019	488	
Clinical indications			
UA (n, %)	577 (28.579)	124 (25.410)	0.290
AMA (n, %)	520 (25.755)	137 (28.074)	0.427
IMSSR (n, %)	506 (25.062)	146 (29.918)	0.096
INIPTR (n, %)	93 (4.606)	13 (2.664)	0.065
Mixed indications (n, %)	81 (4.012)	27 (5.533)	0.157
Other indications (n, %)	242 (11.986)	41 (8.402)	0.043

UA, ultrasound abnormalities; AMA, advanced maternal age; IMSSR, increased maternal serum screening risk; INIPTR, increased NIPT risk. Other indications included previous fetus/child with genetic or phenotypic abnormalities, carriers of monogenic genetic diseases, medication during pregnancy, embryos stop developing, intellectual disability of pregnant women.

^a^Gestational age and maternal age were expressed as mean ± SD.

### Detection of chromosomal abnormalities in the karyotyping and QF-PCR groups

In the karyotyping group, 39 chromosome aneuploidies, 2 triploids, 8 mosaic aneuploidies and 3 derivative chromosomes, were detected (Table 2). Trisomy 21 and sex chromosome aneuploidies were the most common aneuploidies, accounting for nearly 90% of chromosome aneuploidies. Meanwhile, 10 numerical chromosomal abnormalities, including 9 chromosome aneuploidies and 1 mosaic aneuploidy, were detected in the QF-PCR group (Table 2). Consistent with the results in the karyotyping group, sex chromosome aneuploidies and trisomy 21 accounted for nearly 90% of chromosome aneuploidies. Detailed information on the chromosome abnormalities detected in these 2 groups is listed in Table 2. Our results revealed that the detection rates of numerical chromosomal abnormalities were not significantly different between these 2 groups.

**Table 2:** Numerical chromosomal abnormalities and derivative chromosome detected in the karyotyping and QF-PCR group.

	Karyotyping group (n = 2019)	QF-PCR group (n = 488)	P value
Trisomy 21	20 (0.991)	3 (0.615)	0.436
Trisomy 18	4 (0.198)	1 (0.205)	0.977
Sex chromosome aneuploid	15 (0.743)	5 (1.025)	0.536
Triploidies	2 (0.099)	0 (0)	0.486
Mosaic aneuploides	8 (0.396)	1 (0.205)	0.526
Derivative chromosome	3 (0.149)	0 (0)	0.621

Case numbers and percentages for these numbers were listed in the table.

The detection rates of numerical chromosomal abnormalities, including aneuploidies, polyploidies and mosaic aneuploidies, in pregnancies with different indications were different (Table 3). For pregnancies with an increased NIPT risk, the detection rate of aneuploidies and derivative chromosomes was significantly higher than others (22.64%, 24/106). Meanwhile, the detection rates of aneuploidies and triploidies in pregnancies with abnormal ultrasound results (2.00%, 14/701), advanced maternal ages (1.52%, 10/657) and mixed indications (2.78%, 3/108) were significantly higher than those in pregnancies with an increased maternal serum screening risk (0.77%, 5/652). This implied that ultrasound examination and prenatal diagnosis of pregnancies at advanced maternal age improved the detection of chromosome aneuploidies.

**Table 3:** Numerical chromosomal abnormalities in pregnancies with different indications.

Indications	UA (n = 701)	AMA (n = 657)	IMSSR (n = 652)	INIPTR (n = 106)	Mixed indications (n = 108)	Other indications (n = 283)	P value
Trisomy 21	6 (0.86)	3 (0.46)	4 (0.61)	8 (7.55)	2 (1.85)	1 (0.35)	< 0.001
Trisomy 18	1 (0.14)	2 (0.30)	0 (0)	2 (1.89)	0 (0)	0 (0)	0.039
Sex chromosome aneuploidies	4 (0.57)	4 (0.6)	0 (0)	12 (11.32)	1 (0.93)	0 (0)	< 0.001
Triploidies	1 (0.14)	0 (0)	1 (0.15)	0 (0)	0 (0)	0 (0)	0.853
Mosaics	2 (0.29)	1 (0.15)	0 (0)	2 (1.89)	0 (0)	1 (0.35)	0.056
Total	14 (2.00)	10 (1.52)	5 (0.77)	24 (22.64)	3 (2.78)	2 (0.71)	< 0.001

UA, ultrasound abnormalities; AMA, advanced maternal age; IMSSR, increased maternal serum screening risk; INIPTR, increased NIPT risk. Other indications included previous fetus/child with genetic or phenotypic abnormalities, carriers of monogenic genetic diseases, medication during pregnancy, embryos stop developing, intellectual disability of pregnant women. Case numbers and percentages for these numbers were listed in the table.

In addition to unbalanced karyotypic abnormalities, karyotyping was able to detect balanced karyotypic changes and chromosome polymorphisms. In the karyotyping group, translocation (33.33%, 13/39), inversion of chromosome 9 (25.64%, 10/39), extended heterochromatin area (15.38%, 6/39) and polymorphisms in satellites (25.64%, 10/39) were detected in 38 foetuses (Table 4). Because of a fetus carrying 9qh+ and 22pss simultaneously, there were 39 balanced karyotypic abnormalities and chromosomal polymorphisms in 38 foetuses. To investigate the inheritance pattern of these chromosomal changes, parents of 30 foetuses accepted karyotyping. Our results revealed that the vast majority of these karyotypic changes (93.3%, 28/30) were inherited, implying that the combined use of CNV-seq and karyotyping was more suitable for couples carrying balanced karyotypic changes.

**Table 4:** Balanced karyotypic abnormalities and chromosomal polymorphisms detected in the karyotyping group.

Abnormalities	Karyotype	Inheritance pattern
Translocation	46,XX,t(3;21)(q25;q22)	De novo
	46,XX,t(4;6)(p14;p23)	De novo
	45,XY,rob(15;22)(q10;q10)	Maternal
	46,XX,t(10;11)(q22;q21)	Maternal
	46,XX,t(2;8)(p23;q22)	Maternal
	46,XY,t(1;3)(q31;p14)	Maternal
	46,XY,t(10;11)(q24;q24)	Maternal
	46,XY,t(2;10)(p10;q10)	Maternal
	45,XX,rob(15;22)(q10;q10)	Paternal
	45,XY,rob(13;14)(q10;q10)	Paternal
	45,XY,rob(14;21)(q10;q10)	Paternal
	46,XX,t(7;16)(p15;q13)	Paternal
	46,XY,t(1;15)(q42;q11.2)	Unknown
Inversion	46,XX,inv(9)(p12q13)	Maternal
	46,XX,inv(9)(p12q13)	Maternal
	46,XX,inv(9)(p12q13)	Maternal
	46,XX,inv(9)(p12q21)	Maternal
	46,XY,inv(9)(p12q13)	Maternal
	46,XX,inv(9)(p12q13)	Maternal or paternal
	46,XX,inv(9)(p12q13)	Paternal
	46,XX,inv(9)(p12q21)	Paternal
	46,XY,inv(9)(p12q13)	Paternal
	46,XY,inv(9)(p12q13)	Paternal
Extended heterochromatin region	46,XX,1qh+	Paternal
	46,XX,1qh+	Paternal
	46,XY,1qh+	Unknown
	46,XY,1qh+	Unknown
	46,XY,21cenh+	Unknown
Polymorphisms in satellites	46,XX,5ps	Maternal
	46,XY,14pss	Maternal
	46,XX,13pss	Paternal
	46,XX,14pss	Paternal
	46,XY,14ps+	Paternal
	46,XY,21pss	Paternal
	46,XX,13pss	Unknown
	46,XX,15ps+	Unknown
	46,XY,15pss	Unknown
Multiple abnormalities	46,XX,9qh+,22pss	Unknown

### Comparison of reporting time between the karyotyping and QF-PCR groups

**Figure 1:**
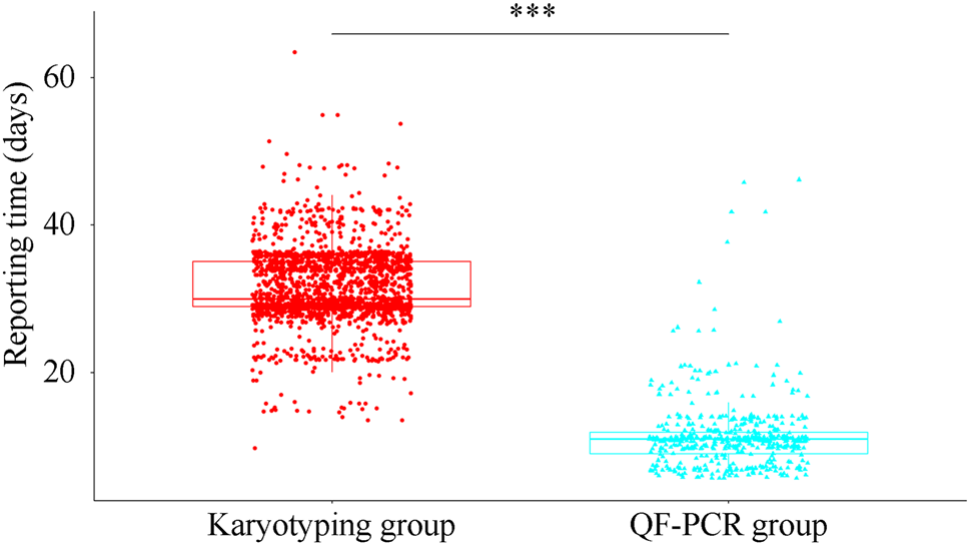
The reporting time of the karyotyping group and QF-PCR group. ***Represented a P < 0.001.

**Figure 2:**
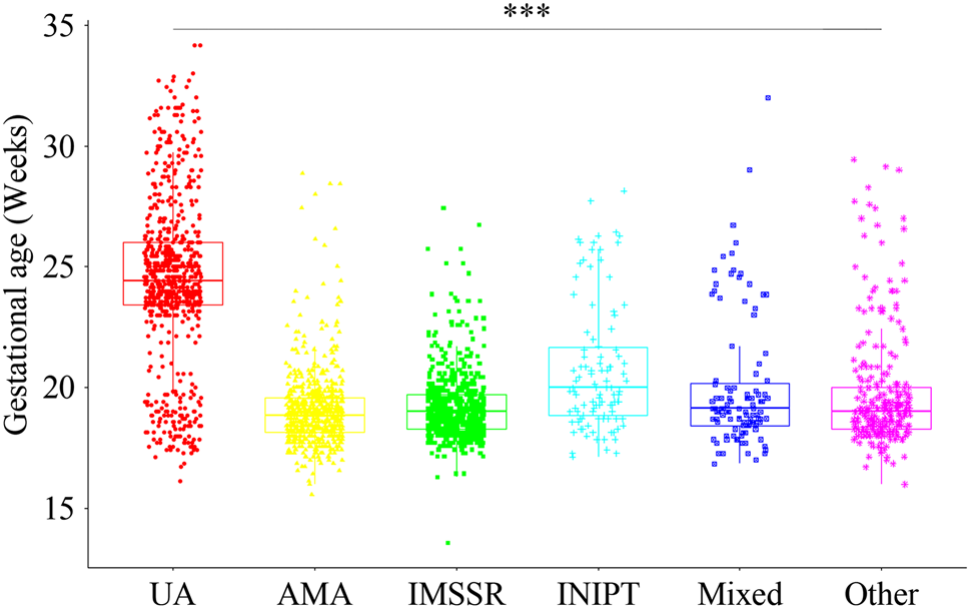
The gestational weeks of pregnancies with different indications. UA, ultrasound abnormalities; AMA, advanced maternal age; IMSSR, increased maternal serum screening risk; INIPTR, increased NIPT risk. Other indications included previous fetus/child with genetic or phenotypic abnormalities, carriers of monogenic genetic diseases, medication during pregnancy, embryos stopping developing, and intellectual disability of pregnant women. ***Represented a P < 0.001.

The turnaround time was compared between these 2 strategies. Theoretically, the reporting time of the combined CNV-seq and karyotyping or QF-PCR was approximately 3 weeks and 1–2 weeks, respectively. Based on these timings, we expected that the reporting time for the karyotyping group would be much longer than that of the QF-PCR group. Consistent with this assumption, our results revealed that QF-PCR reduced the reporting time from 31.593 ± 4.944 days to 11.460 ± 4.894 days (Fig. 1). The gestational ages of participants with different indications were also analyzed. Our results revealed that pregnancies with ultrasound abnormalities were at more advanced gestational ages than others (Fig. 2). For these pregnancies, the combined use of QF-PCR and CNV-seq was a better choice than the combined use of karyotyping and CNV-seq.

### Detection of CNVs in pregnancies with different indications

**Figure 3:**
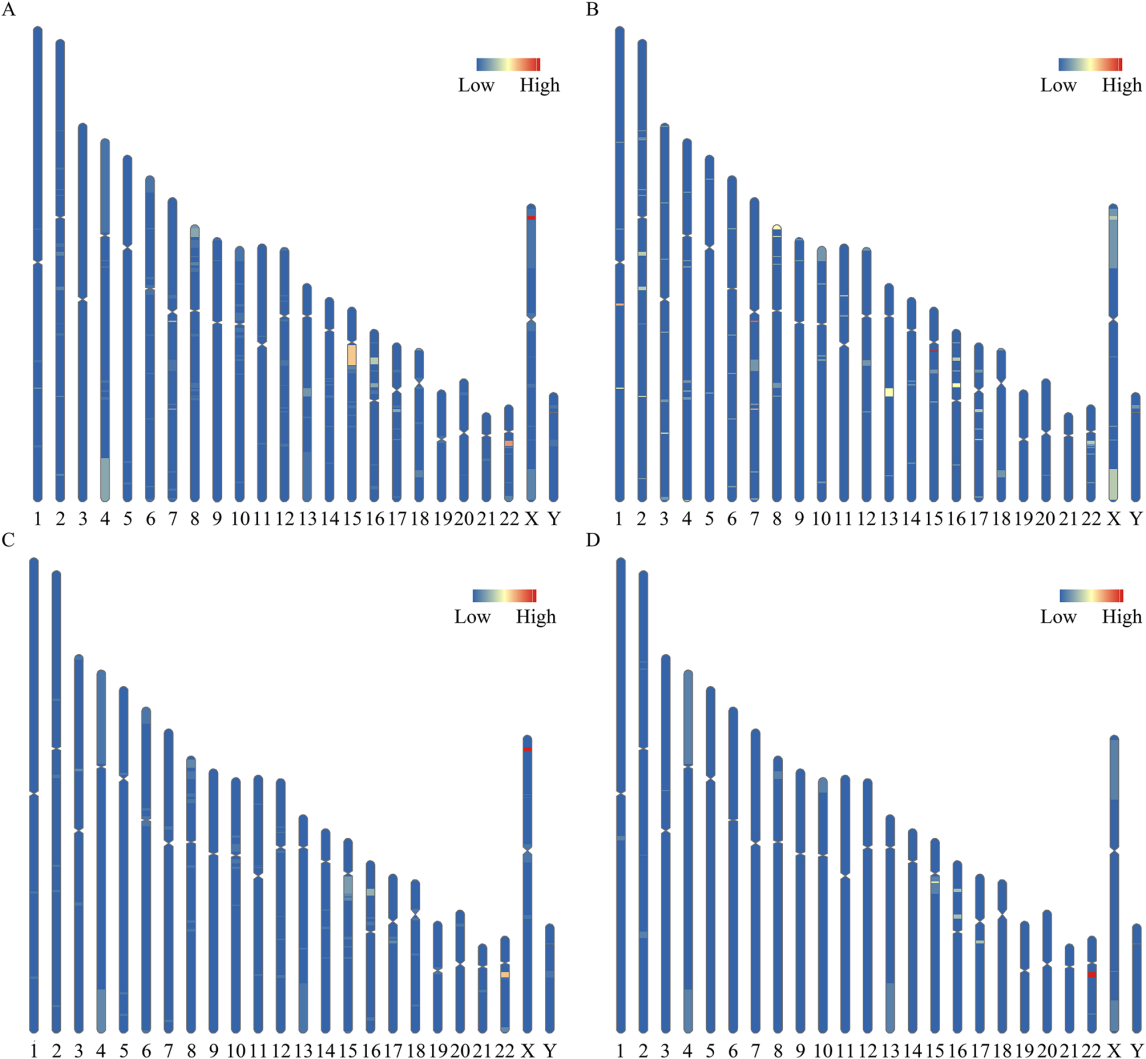
The distribution of CNVs on each chromosome. Schematic diagrams of the distribution of all CNVs (**A**), microdeletions (**B**), microduplications (**C**) and pCNVs (**D**) detected in our study. Blue areas represented that no CNVs were detected in that area. The more CNVs detected in an area, the area was redder.

With the use of CNV-seq, CNVs were effectively detected in both groups. As a result, a total of 253 CNVs, including 133 microdeletions and 120 microduplications, were detected in 233 foetuses (Fig. 3A, Supplement Table 1). Most foetuses (91.42%, 213/233) carried only one CNV and the average length of CNV detected in our study was 1.88 Mb. Among these microdeletions, 24, 3 and 106 were classified as pathogenic, likely pathogenic and VUS, respectively. Meanwhile, 16, 1 and 103 microduplications were classified as pathogenic, likely pathogenic and VUS, respectively. The distribution tendencies of microdeletions and microduplications were different. Microdeletions were more likely to occur on chromosomes 1, 7 and 15, while microduplications were more likely to occur on chromosomes 15, 22 and X (Fig. 3B and C).

The detection rates of CNVs in pregnancies with different indications were also analyzed. Although the detection rates of VUS in pregnancies with different indications were not significantly different, pCNVs and likely pathogenic CNVs (lpCNVs) were more likely to be identified in pregnancies with increased NIPT risk, increased maternal serum screening risk and ultrasound abnormalities (Table 5). As shown in Fig. 3D, pCNVs and lpCNVs were more likely to occur on chromosomes 22, 15 and 16. Subsequent analysis revealed that the incidence of pCNVs and lpCNVs varied in pregnancies with different indications and microduplication of 22q11.21 and microdeletion of 15q11.2 were the most common pCNVs (Table 6).

**Table 5:** Detection of CNVs in pregnancies with different indications.

Indications	Sample number	CNVs (n, %)	pCNVs and lpCNVs (n, %)	VOUS (n, %)
UA	701	68 (9.700)	12 (1.712)	56 (7.989)
AMA	657	46 (7.002)	2 (0.304)	44 (6.697)
IMSSR	652	78 (11.963)	15 (2.301)	63 (9.663)
INIPTR	106	21 (19.811)	12 (11.321)	9 (8.491)
Mixed indications	108	10(9.260)	1 (0.926)	9 (8.333)
Other indications	283	30 (10.601)	2 (0.707)	28 (9.894)
P value	–	0.005	< 0.001	0.521

UA, ultrasound abnormalities; AMA, advanced maternal age; IMSSR, increased maternal serum screening risk; INIPTR, increased NIPT risk; lpCNVs, likely pathogenic CNVs. Other indications included previous fetus/child with genetic or phenotypic abnormalities, carriers of monogenic genetic diseases, medication during pregnancy, embryos stop developing, intellectual disability of pregnant women.

**Table 6:** Detailed information of pCNS and lpCNVs in the karyotyping and QF-PCR group.

Number	Indications	Maternal Age (years)	Gestational Age (weeks)	Karyotype	CNV results (GRCh37)	CNV size (Mb)	Dosage sensitivity area/gene involved	Classification
1	UA	24	23.43	46,XY	chr22:g.18950001_21500004del	2.55	22q11 deletion syndrome	P
2	UA	28	27.14	46,XY	chr22:g.40500005_41050004del	0.55	TNRC6B (HI Score: 3)	P
3	UA	27	18.86	46,XX	chr8:g.8110001_11960000dup	3.85	8p23.1 duplication syndrome	P
4	UA	27	24.43	46,XX	chr1:g.145810001_147910000del	2.1	1q21.1 recurrent region (BP3-BP4, distal) (includes GJA5)	P
5	UA	23	19.43	46,XY	chr15:g.22776624_23076623del	0.3	15q11.2 recurrent region (BP1-BP2) (includes NIPA1)	P
6	UA	26	24.86	46,XX	chr15:g.22776624_23276623del	0.5	15q11.2 recurrent region (BP1-BP2) (includes NIPA1)	P
7	UA	26	25.00	46,XY	chr16:g.14860001_16410000del	1.55	16p13.11 recurrent region (BP2-BP3) (includes MYH11)	P
8	UA	31	18.86	46,XY	chr16:g.14860001_16610000del	1.75	16p13.11 recurrent region (BP2-BP3) (includes MYH11)	P
9	UA	23	30.14	46,XY	chr16:g.28760001_29060000del	0.3	16p11.2 recurrent region (distal, BP2-BP3) (includes SH2B1)	P
10	UA	26	27.00	46,XY	chr17:g.34800001_36250000del	1.45	RCAD (renal cysts and diabetes)	P
11	UA	33	24.86	46,XX	chr2:g.47587852_47987851del	0.4	MSH2 (HI Score: 3)	P
12	UA	25	20.71	46,XY	chr3:g.71010005_71410004del	0.4	FOXP1 (HI Score: 3)	LP
13	AMA	34	18.00	46,XY	chr2:g.148829852_149029851del	0.2	MBD5 (HI Score: 3)	LP
14	AMA	39	28.43	46,XY	chr2:g.51237852_51587851del	0.35	NRXN1 (HI Score: 3)	LP
15	IMSSR	29	19.71	46,XY	chr16:g.29710001_30210000dup	0.5	16p11.2 microduplication syndrome	P
16	IMSSR	29	18.86	46,XY	chr15:g.22676624_23226623del	0.55	15q11.2 recurrent region (BP1-BP2) (includes NIPA1)	P
17	IMSSR	29	18.57	46,XX	chr15:g.22776624_23076623del	0.3	15q11.2 recurrent region (BP1-BP2) (includes NIPA1)	P
18	IMSSR	28	18.00	46,XX	chr16:g.14860001_16460000dup	1.6	16p13.11 recurrent microduplication (neurocognitive disorder susceptibility locus)	LP
19	IMSSR	29	18.14	46,XX	chr22:g.18850001_21450004dup	2.6	22q11 duplication syndrome	P
20	IMSSR	24	19.86	46,XX	chr22:g.18900001_21550004dup	2.65	22q11 duplication syndrome	P
21	IMSSR	31	17.86	46,XX	chrX:g.123460001_123760000del	0.3	SH2D1A (HI Score: 3)	P
22	IMSSR	29	18.00	46,XY	chrX:g.6410001_8160000del	1.75	Xp22.31 recurrent region (includes STS)	P
23	IMSSR	26	17.86	46,XX	chrX:g.6960001_7310000del	0.35	STS (HI Score: 3)	P
24	IMSSR	27	18.00	46,XX	chr13:g.100520001_100870000del	0.35	ZIC3 (HI Score: 3)	P
25	IMSSR	32	20.71	46,XY	chr15:g.20000001_28976623dup	8.977	15q11.2q13 recurrent (PWS/AS) region (Class 1, BP1-BP3)	P
26	IMSSR	30	18.43	46,XX	chr16:g.28310001_30310000del	2	16p11.2 recurrent region (distal, BP2-BP3) (includes SH2B1)	P
27	IMSSR	27	19.00	46,XX	chr17:g.34800001_36350000dup	1.55	17q12 recurrent (RCAD syndrome) region (includes HNF1B)	P
28	IMSSR	22	19.29	46,XY	chr22:g.18850001_21500004dup	2.65	22q11 duplication syndrome	P
29	IMSSR	31	19.14	46,XX	chr22:g.18850001_21600004dup	2.75	22q11 duplication syndrome	P
30	INIPTR	23	22.43	46,XX	chr4:g.10001_49057700dup	49.048	4p trisomy syndrome	P
31	INIPTR	35	23.43	46,XX	chrX:g.138910001_154960000del	16.05	Xq28 recurrent region (int22h1/int22h2-flanked) (includes RAB39B)	P
32	INIPTR	24	19.43	46,XX	chr13:g.88370001_115070000dup; chrX:g.2610001_33610000del	26.7; 31	13q trisomy syndrome; Xp22.31 recurrent region (includes STS)	P
33	INIPTR	27	17.86	47,XXY	chr15:g.22676624_23276623del	0.6	15q11.2 recurrent region (BP1-BP2) (includes NIPA1)	P
34	INIPTR	30	21.29	46,XX	chr2:g.189079852_192329851del	3.25	COL3A1 (HI Score: 3)	P
35	INIPTR	21	20.00	46,XY	chr22:g.18850001_21450004dup	2.6	22q11 duplication syndrome	P
36	INIPTR	26	19.86	46,XX	chr22:g.18850001_21500004dup	2.65	22q11 duplication syndrome	P
37	INIPTR	35	18.71	46,XX	chr22:g.18850001_21500004dup	2.65	22q11 duplication syndrome	P
38	INIPTR	23	21.57	46,XY	chr22:g.18900001_21450004del	2.55	22q11 deletion syndrome	P
39	INIPTR	29	20.00	46,XX	chr22:g.18900001_21450004dup	2.55	22q11 duplication syndrome	P
40	INIPTR	23	18.00	46,XX	chr4:g.167482601_191032600dup	23.55	none	P
41	Mixed indications	31	17.29	46,XY	chr9:g.138660001_140160000del	1.5	none	P
42	Other indications	34	26.00	46,XX	chr10:g.60001_7660000del	7.6	ZMYND11 (HI Score: 3)	P
43	Other indications	31	17.43	46,XY	chr17:g.34800001_36250000dup	1.45	17q12 recurrent (RCAD syndrome) region (includes HNF1B)	P

UA, ultrasound abnormalities; AMA, advanced maternal age; IMSSR, increased maternal serum screening risk; INIPTR, increased risk of NIPT; P, pathogenic; lp, likely pathogenic CNVs. Other indications included previous fetus/child with genetic or phenotypic abnormalities, carriers of monogenic genetic diseases, medication during pregnancy, embryos stop developing, intellectual disability of pregnant women.

To investigate the inheritance pattern of CNVs detected in our study, CNV-seq was performed for parents of 123 foetuses. Because of 9 foetuses carrying 2 CNVs simultaneously, a total of 132 CNVs were detected in 123 foetuses. Subsequent results revealed that 46, 71 and 15 of these CNVs were inherited from their father, their mother and de novo, respectively (Supplement Table 2). The inheritance pattern of pCNVs, lpCNVs and VUS was further analyzed. Among 21 pCNVs and lpCNVs, 17 and 4 were inherited from their mother and de novo, respectively. Meanwhile, among 111 VUS, 46, 54 and 11 were inherited from their father, their mother and de novo, respectively. Although VUS were more likely to be inherited, the difference in the ratio of inherited CNVs was not statistically significant between VUS and pCNVs, lpCNVs (P = 0.226). In conclusion, our results revealed that the majority of CNVs detected in foetuses were inherited.

## Discussion

To systematically compare the combined use of CNV-seq and karyotyping and the combined use of CNV-seq and QF-PCR, a total of 2507 pregnant women were included in our study and divided into a karyotyping group and QF-PCR group. The most common indications in our study were ultrasound abnormalities, advanced maternal age and increased maternal serum screening risk, accounting for approximately 80% of all pregnancies. In our study, the most common unbalanced karyotypic change was trisomy 21 (37.10%, 23/62), followed by sex chromosome aneuploidies (32.26%, 20/62), mosaic aneuploidies (14.52%, 9/62), trisomy 18 (8.06%, 5/62), derivative chromosome (4.84%, 3/62) and triploidies (3.23%, 2/62). The detection rates of these karyotypic abnormalities were not significantly different between these 2 groups, revealing that both strategies were able to detect unbalanced karyotypic changes effectively. Compared with previous reports^19,20^, the detection rate of trisomy 21 and sex chromosome abnormalities decreased and increased significantly in our study, respectively. As revealed by Zhao et al.^21^, NIPT was an effective method for prenatal screening of sex chromosome aneuploidies. Including more pregnancies with increased NIPT risk in our study may lead to this disparity. In addition to numerical chromosomal abnormalities, the combined use of CNV-seq and karyotyping was also able to detect balanced karyotypic changes and chromosome polymorphisms. In our study, the most common balanced karyotypic abnormality was translocation (33.33%, 13/39), followed by inversion of chromosome 9 (25.64%, 10/39), polymorphisms in satellites (25.64%, 10/39) and extended heterochromatin area (15.38%, 6/39). Consistent with previous reports^22,23^, we found that most of these balanced karyotypic changes and chromosome polymorphisms were inherited. Therefore, parental karyotyping is vital for choosing a supplementary method of CNV-seq in prenatal diagnosis. For couples carrying balanced karyotypic changes, using karyotyping and CNV-seq was a better choice.

Turnaround time was an important factor to take into account when choosing prenatal diagnostic strategies. For pregnant women, especially those at advanced gestational ages, shorter turnaround time helped to relieve maternal anxiety and gave them more time for post-test counseling and interventions. Therefore, the turnaround time was also compared between these 2 strategies. Our results revealed that QF-PCR significantly reduced the turnaround time. Interestingly, consistent with previous reports^24^, our results revealed that pregnancies with abnormal ultrasound results were usually at more advanced gestational ages. For these women, a shorter turnaround time helped to relieve maternal anxiety and provided more time for further interventions. Therefore, for pregnancies at advanced gestational age, such as those with ultrasound abnormalities, QF-PCR was a better supplement to CNV-seq.

Although the detailed mechanism remained elusive, microdeletions and microduplications occurred in different hot spots. Subsequent analysis revealed that pCNVs and lpCNVs were more likely to occur on chromosomes 22, 15 and 16. Although previous reports showed that the Xp22.31 microdeletion and 22q11.2 microdeletion were the most common pCNVs^13,19^, the microdeletion of 15q11.2 and microduplication of 22q11.21 were the most prevalent pCNVs in our study. Interestingly, most microduplications of 22q11.21 were detected in pregnancies with increased NIPT risk. Therefore, including more pregnancies with increased NIPT risk in our study may elevate the detection rate of microduplication of 22q11.21. Additionally, conducting research in different districts may also lead to this inconsistency. The inheritance pattern of CNVs detected in our study was also investigated. Although the ratio of inherited CNVs was not statistically different between pCNVs, lpCNVs and VUS, our results hinted that pCNVs and lpCNVs were more likely to be de novo. Future research with a larger sample size may elucidate the inheritance pattern of CNVs with different pathogenicity.

NIPT, maternal serum screening and prenatal ultrasound examination are routinely used for prenatal detection of foetal abnormalities. The detection of chromosome abnormalities and pCNVs in pregnancies with different indications was analysed in our study. Growing evidence has revealed that NIPT dramatically improves the detection of common aneuploidies and pCNVs^25,26^. Consistent with these reports, the detection rates of aneuploidies and pCNVs in pregnancies with an increased NIPT risk were elevated significantly in our study. Moreover, compared with previous reports, including more pregnancies with an increased NIPT risk elevated the detection rates of sex chromosome aneuploidies and microduplication of 22q11.2^19,20^. Maternal serum screening has been used for prenatal screening of foetal aneuploidies since the 1950s^27^. Recent evidence has shown that abnormal serum screening results are associated with foetal pCNVs^28^. In our study, although the detection rates of aneuploidies in pregnancies with an increased maternal serum screening risk were not significantly increased, the detection rates of pCNVs and lpCNVs in these pregnancies elevated significantly. Growing evidence revealed that the detection rates of foetal aneuploidies and pCNVs in pregnancies with ultrasound abnormalities increased significantly^12,24,29,30^, and CNV-seq was an effective way to detect foetal pCNVs in pregnancies with ultrasound abnormalities^24^. Consistent with these reports, our results revealed that the detection rates of foetal karyotypic anomalies and pCNVs in pregnancies with ultrasound abnormalities was increased. Taken together, although the detection efficiency of chromosomal abnormities and pCNVs varied between different screening methods, our results revealed that systematic prenatal examinations improved the detection of genetic abnormalities in foetuses and reduced the incidence of birth defects.

In conclusion, the combined use of CNV-seq and karyotyping was able to detect balanced karyotypic changes and chromosome polymorphisms, while the combined use of CNV-seq and QF-PCR was able to detect maternal cell contamination and significantly reduced the turnaround time. Therefore, parental karyotyping is important in selecting a supplementary method of CNV-seq. For couples carrying balanced karyotypic changes, karyotyping was a better supplement for CNV-seq. For couples with normal karyotypes and pregnant women at advanced gestational ages, the combined use of CNV-seq and QF-PCR was the best option studied. Moreover, our results revealed that NIPT, maternal serum screening and prenatal ultrasound scans improved the detection rates of chromosome anomalies and pCNVs.

## Materials and methods

### Study design and participants

A total of 2507 pregnant women within the 13^+4th^ to 34^+1th^ gestational weeks were included in our study from September 2019 to December 2021 at the Deyang People’s Hospital. All pregnancies were singleton. Indications for prenatal diagnosis were ultrasound abnormalities, advanced maternal age (> 35 years), increased maternal serum screening risk, increased non-invasive prenatal testing (NIPT) risk, mixed indications and other indications. Ultrasound abnormalities included increased fetal nuchal translucency (NT) thickness, nasal bone hypoplasia, short femur length, thicken nuchal fold, ventriculomegaly, echogenic bowel, echogenic intracardiac focus, choroid plexus cysts, aberrant right subclavian artery and single umbilical artery. Mixed indications were pregnant women with two or more indications. Other indications included previous fetus/child with genetic or phenotypic abnormalities, carriers of monogenic genetic diseases, medication during pregnancy, embryos stopping developing, and intellectual disability of pregnant women. Detailed clinical information of the participants is listed in Table 1. According to prenatal diagnosis strategies, participants were divided into a QF-PCR group and a karyotyping group. In the karyotyping group, 2019 participants chose to accept CNV-seq and karyotyping. CNV-seq and QF-PCR were performed for 488 participants in the QF-PCR group. All methods were performed following the relevant guidelines and regulations in our study. This study was approved by the Ethical Committee of Deyang People’s Hospital, and informed consent was obtained from all participants.

### QF-PCR assay

Rapid detection of common foetal chromosome aneuploidy was performed using the STR Genotyping Kit for Chromosomes 13/18/21/X/Y (DaRui Biotech Co., Guangzhou, China) according to the manufacturer’s instructions. Briefly, the experimental process included genomic DNA extraction, PCR amplification and capillary electrophoresis. Genomic DNA was extracted from 8 ml of amniotic fluid using the TIANamp Genomic DNA kit (Tiangen Biotech Co., Beijing, China) according to the manufacturer’s instructions. The concentrations of genomic DNA were measured using Qubit 1 × dsDNA High Sensitivity (HS) and Broad Range (BR) Assay Kits (Thermo Fisher Scientific Inc., Rockford, USA). Subsequently, the concentration of genomic DNA was diluted to 5–10 ng/μl. PCR amplification of 20 highly polymorphic short tandem repeats (STRs) (Table 7) was performed using a Bio–Rad PTC 200 PCR system (Bio–Rad Laboratories, Hercules, USA). The PCR profile was pre-denaturation at 95 °C for 5 min followed by 95 °C for 30 s, 58 °C for 40 s, and 72 °C for 50 s, for 25 cycles with a final extension at 72 °C for 10 min. After PCR amplification, 1 μl PCR products were mixed with 13.5 μl HiDi formamide (Thermo Fisher) and 1 μl LIZ600 (Thermo Fisher), and then capillary electrophoreses were performed using the 3500 ABI Genetic Analyser (Applied Biosystems, Waltham, USA). Finally, the electrophoresis results were interpreted according to the manufacturer’s instructions.

**Table 7:** STR markers analyzed by QF-PCR.

Chromosome	STR	Length (bp)
21	21q11.2	170–220
21	DS21S1411	270–325
21	DS21S1412	380–450
21	DS21S1414	315–370
21	DS21S1433	140–190
21	DS21S1445	470–530
18	DS18S1002	108–140
18	DS18S386	305–375
18	DS18S391	180–220
18	DS18S535	235–280
13	DS13S305	370–430
13	DS13S628	140–190
13	DS13S634	320–365
13	DS13S742	220–275
X/Y	AMXY	102/108
X	DXS1187	130–170
X	DXS6809	255–300
X	DXS8377	180–254
X	DXS981	310–370
Y	SRY	248

### CNV-seq

CNV-seq was performed to detect chromosome aneuploidy and pCNVs. The workflow of CNV-seq included extracting genomic DNA, constructing a library, quality control, pooling, sequencing, bioinformatics analysis and interpreting the results. First, genomic DNA was extracted from 2 to 4 ml of amniotic fluid using the TIANamp Genomic DNA kit (Tiangen) according to the manufacturer’s instructions. Then, 20 ng genomic DNA was fragmented by NEBNext dsDNA Fragmentase (New England Biolabs, Ipswich, USA) at 37 °C for 50 min. Subsequently, end filling, adaptor ligation and PCR amplification were conducted to construct a DNA library using Foetal Chromosome Aneuploidy (T21, T18, and T13) Testing Kits (Annoroad, Beijing, China). The concentrations of the libraries were measured by Qubit 1X dsDNA High Sensitivity (HS) and Broad Range (BR) Assay Kits (Thermo Fisher). Agilent 2100 Bioanalyzed (Agilent Technologies, Palo Alto, USA) was also used for quality control of libraries. Subsequently, qualified libraries were pooled together and subjected to massively parallel sequencing using NextSeq 550AR (Illumina, San Diego, USA). For each sample, at least 4.5 million raw reads with a length of 40 bp were generated for further analysis. The quality control criteria of the sequencing results were as follows: reads > 4.5 Mb, GC content: 38.5–45.5%, Q30 ratio > 85%, alignment ratio > 62.5%, unique read ratio > 60%, and duplication ratio < 10%. Qualified sequencing results were mapped to the grch37 version of the human genome using the Burrows–Wheelers algorithm^31^, and copy number variations (CNVs) were detected by bioinformatics analysis.

The pathogenicity of CNVs was classified as pathogenic, likely pathogenic, variant of uncertain significance (VUS), likely benign and benign according to American College of Medical Genetics guidelines^32^. Public databases, including ClinGen, Database of Genomic Variation and Phenotype in Humans Using Ensembl Resources (DECIPHER), Online Mendelian Inheritance in Man (OMIM), 1000 Genomes, and the Database of Genomic Variants, were used to interpret the pathogenicity of these CNVs. CNVs were described according to ISCN 2020 guidelines.

### Karyotyping

A total of 20 ml amniotic fluid was collected from each pregnant woman and then equally divided into 2 parts. After centrifugation at 1000 rpm for 10 min, the supernatant was discarded. Then, precipitated amniocytes were resuspended in 3 ml BIO-AMF-3 complete medium (Biological Industries, Cromwell, USA) and incubated at 37 °C in a Thermo 3111 CO_2_ incubator (Thermo Fisher). For each sample, 2 independent cultures were established. Subsequently, amniocytes were harvested for G banding after culturing for 9–14 days. For each sample, 20 metaphase images were captured and counted using a Zeiss automatic karyotyping scanning system (Carl Zeiss, Jena, Germany). Among these metaphases, 5 were analysed using IKAROS software (Carl Zeiss). Karyotypes were described according to ISCN 2020 guidelines.

### Statistics

Statistical analysis was performed using SPSS 19.0 (IBM, New York, USA). Qualitative data were analysed by the chi-square test, while normally distributed quantitative data were analysed by t-test and one-way ANOVA. A P < 0.05 indicated that the difference was statistically significant. The schematic diagrams of distributions of CNVs on chromosomes and boxplots were drawn using R packages RIdeogram^33^ and ggplot2, respectively.

## Supplementary Information

Supplementary Table 1.

Supplementary Table 2.

## Data Availability

The datasets used and/or analyzed during this study are available from the corresponding author upon reasonable request.
